# Supplementary material for: HIVprotI: an integrated web based platform for prediction and design of HIV proteins inhibitors
Source: J Cheminform. 2018 Mar 9;10:12. doi: 10.1186/s13321-018-0266-y (PMC5845081; doi:10.1186/s13321-018-0266-y)
Supplement: Supplementary file 2 — Additional file 2. Source code of HIVProtI web server. [file 13321_2018_266_MOESM2_ESM.zip › HIVprotI_Source-code/hivprotI/weka-3-6-6/doc/weka/core/pmml/Expression.html]

Expression


---


|  |  |  |
| --- | --- | --- |
| **Overview** \| **Package** \| **Class** \| **Tree** \| **Deprecated** \| **Index** \| **Help** \| **Weka's home** | |  |
| **PREV CLASS**   **NEXT CLASS** | **FRAMES**    **NO FRAMES**     **All Classes** |  |
| SUMMARY: NESTED \| FIELD \| CONSTR \| METHOD | DETAIL: FIELD \| CONSTR \| METHOD |  |


---


## weka.core.pmml Class Expression

```
java.lang.Object
  weka.core.pmml.Expression
```

**All Implemented Interfaces:**: java.io.Serializable

**Direct Known Subclasses:**: Constant, Discretize, FieldRef, NormContinuous, NormDiscrete

---

``` public abstract class Expression extends java.lang.Object implements java.io.Serializable ```

**See Also:**: Serialized Form

---

**Constructor Summary**

`Expression(FieldMetaInfo.Optype opType,
java.util.ArrayList<Attribute> fieldDefs)`


| **Method Summary** | |
| --- | --- |
| `static Expression` | `getExpression(org.w3c.dom.Node container, FieldMetaInfo.Optype opType, java.util.ArrayList<Attribute> fieldDefs, weka.core.pmml.TransformationDictionary transDict)`             Static factory method that returns a subclass of Expression that encapsulates the type of expression contained in the Element supplied. |
| `static Expression` | `getExpression(java.lang.String name, org.w3c.dom.Node expression, FieldMetaInfo.Optype opType, java.util.ArrayList<Attribute> fieldDefs, weka.core.pmml.TransformationDictionary transDict)`             Static factory method that returns a subclass of Expression that encapsulates the type of expression supplied as an argument. |
| `Attribute` | `getFieldDef(java.lang.String attName)`             Return the named attribute from the list of reference fields. |
| `int` | `getFieldDefIndex(java.lang.String attName)` |
| `FieldMetaInfo.Optype` | `getOptype()`             Get the optype of the result of applying this Expression. |
| `abstract  double` | `getResult(double[] incoming)`             Get the result of evaluating the expression. |
| `abstract  java.lang.String` | `getResultCategorical(double[] incoming)`             Gets the result of evaluating the expression when the optype is categorical or ordinal as the actual String value. |
| `double` | `getResultContinuous(double[] incoming)`             Get the result of evaluating the expression for continuous optype. |
| `void` | `setFieldDefs(java.util.ArrayList<Attribute> fieldDefs)`             Set the field definitions for this Expression to use |
| `java.lang.String` | `toString()` |
| `java.lang.String` | `toString(java.lang.String pad)` |

**Methods inherited from class java.lang.Object**

`equals, getClass, hashCode, notify, notifyAll, wait, wait, wait`

**Constructor Detail**

### Expression

```
public Expression(FieldMetaInfo.Optype opType,
                  java.util.ArrayList<Attribute> fieldDefs)
```


**Method Detail**

### setFieldDefs

```
public void setFieldDefs(java.util.ArrayList<Attribute> fieldDefs)
                  throws java.lang.Exception
```

:   Set the field definitions for this Expression to use

    :   **Parameters:**: `fieldDefs` - the field definitions to use **Throws:**: `java.lang.Exception` - if there is a problem setting the field definitions

---


### getResult

```
public abstract double getResult(double[] incoming)
                          throws java.lang.Exception
```

:   Get the result of evaluating the expression. In the case
    of a continuous optype, a real number is returned; in
    the case of a categorical/ordinal optype, the index of the nominal
    value is returned as a double.

    :   **Parameters:**: `incoming` - the incoming parameter values **Returns:**: the result of evaluating the expression **Throws:**: `java.lang.Exception` - if there is a problem computing the result

---


### getResultContinuous

```
public double getResultContinuous(double[] incoming)
                           throws java.lang.Exception
```

:   Get the result of evaluating the expression for continuous
    optype. Is the same as calling getResult() when the optype
    is continuous.

    :   **Parameters:**: `incoming` - the incoming parameter values mining schema **Returns:**: the result of evaluating the expression. **Throws:**: `java.lang.Exception` - if the optype is not continuous.

---


### getResultCategorical

```
public abstract java.lang.String getResultCategorical(double[] incoming)
                                               throws java.lang.Exception
```

:   Gets the result of evaluating the expression when the
    optype is categorical or ordinal as the actual String
    value.

    :   **Parameters:**: `incoming` - the incoming parameter values **Returns:**: the result of evaluating the expression **Throws:**: `java.lang.Exception` - if the optype is continuous

---


### getExpression

```
public static Expression getExpression(org.w3c.dom.Node container,
                                       FieldMetaInfo.Optype opType,
                                       java.util.ArrayList<Attribute> fieldDefs,
                                       weka.core.pmml.TransformationDictionary transDict)
                                throws java.lang.Exception
```

:   Static factory method that returns a subclass of Expression that
    encapsulates the type of expression contained in the Element
    supplied. Assumes that there is just one expression contained
    in the supplied Element.

    :   **Parameters:**: `container` - the Node containing the expression: `opType` - the optype of the value returned by this Expression.: `fieldDefs` - an ArrayList of Attributes for the fields that this Expression may need to access Since Expressions are children of either DerivedFields or DefineFuntions, they will have the same optype as their parent.: `transDict` - the TransformationDictionary (may be null if there is no dictionary) **Returns:**: an Expression object or null if there is no known expression in the container **Throws:**: `java.lang.Exception` - for unsupported Expression types

---


### getExpression

```
public static Expression getExpression(java.lang.String name,
                                       org.w3c.dom.Node expression,
                                       FieldMetaInfo.Optype opType,
                                       java.util.ArrayList<Attribute> fieldDefs,
                                       weka.core.pmml.TransformationDictionary transDict)
                                throws java.lang.Exception
```

:   Static factory method that returns a subclass of Expression that
    encapsulates the type of expression supplied as an argument.

    :   **Parameters:**: `name` - the name of the Expression to get: `expression` - the Node containing the expression: `opType` - the optype of the value returned by this Expression.: `fieldDefs` - an ArrayList of Attributes for the fields that this Expression may need to access Since Expressions are children of either DerivedFields or DefineFuntions, they will have the same optype as their parent.: `transDict` - the TransformationDictionary (may be null if there is no dictionary) **Returns:**: an Expression object or null if there is no known expression in the container **Throws:**: `java.lang.Exception` - for unsupported Expression types

---


### getFieldDef

```
public Attribute getFieldDef(java.lang.String attName)
```

:   Return the named attribute from the list of reference fields.

    :   **Parameters:**: `attName` - the name of the attribute to retrieve **Returns:**: the named attribute (or null if it can't be found).

---


### getFieldDefIndex

```
public int getFieldDefIndex(java.lang.String attName)
```

---


### getOptype

```
public FieldMetaInfo.Optype getOptype()
```

:   Get the optype of the result of applying this Expression.

    :   **Returns:**: the optype of the result of applying this Expression

---


### toString

```
public java.lang.String toString()
```

:   **Overrides:**: `toString` in class `java.lang.Object`

---


### toString

```
public java.lang.String toString(java.lang.String pad)
```


---


|  |  |  |
| --- | --- | --- |
| **Overview** \| **Package** \| **Class** \| **Tree** \| **Deprecated** \| **Index** \| **Help** \| **Weka's home** | |  |
| **PREV CLASS**   **NEXT CLASS** | **FRAMES**    **NO FRAMES**     **All Classes** |  |
| SUMMARY: NESTED \| FIELD \| CONSTR \| METHOD | DETAIL: FIELD \| CONSTR \| METHOD |  |


---
